# Utility of Opportunistic Infections, Joints’ Involvement and Accuracy of Various Screening Tests to Diagnose Rheumatoid Arthritis Patients

**DOI:** 10.3390/medicina59020367

**Published:** 2023-02-14

**Authors:** Ausaf Ahmad, Ashfaque Khan, Abdur Raheem Khan, Hashim Ahmed, Ramzi Abdu Alajam, Mohammed M. Alshehri, Bhuvanesh Babu Mondey Ganesan, Salma Jabril Abu Dayah, Mohammad Abu Shaphe, Abdulaziz H. Alameer, Vandana Esht

**Affiliations:** 1 Department of Community Medicine, IIMS&R, Integral University, Lucknow 226026, India; 2 Department of Physiotherapy, Integral University, Lucknow 226026, India; 3 Department of Medical Rehabilitation Science, College of Applied Medical Sciences, Najran University, Najran 55461, Saudi Arabia; 4 Department of Physical Therapy, College of Applied Medical Sciences, Jazan University, Jazan 45142, Saudi Arabia

**Keywords:** rheumatoid arthritis, sign symptoms, joint pain

## Abstract

*Background and Objectives*: Rheumatoid Arthritis (RA) is an auto-immune disease in which the body mistakenly considers some parts of its own system as pathogens and attacks them. Prevalence is approximately 0.75% in India. About 40% of the diseased become work disabled within 5 years from the onset of symptoms. The objective of this paper is to assess the sign/symptoms, joints’ involvement, difficulties in daily activities and screening accuracy of serology tests of clinically suspected RA patients. *Material and Methods*: A cross-sectional cohort study design was conducted on two hundred ninety clinically suspected subjects who were referred by different OPDs of hospitals for screening. The profiles of study subjects were carried through a semi-structured, pre-tested schedule method. About 2 mL of blood samples were collected in a plain vial from each patient and tested for diagnostic tests RF, CRP and AntiCCP by using RF-Latex, CRP Latex and ELISA method, respectively, by the laboratory persons. *Results*: Joint pain shows to be a leading problem in RA as compared to other signs and symptoms. The majority of the study subjects suffer from knee problems (62%). Approximately equal numbers of RA-positive cases were screened by RF and AntiCCP tests. The CRP test screened about one-third of cases. CRP+ AntiCCP, RF+ AntiCCP and RF + CRP all have good sensitivity, and RF+ AntiCCP + CRP has a very high sensitivity for diagnosing RA. *Conclusions*: This study found that a substantiation of a major proportion of clinically suspected RA patients were suffering from knee pain. Predication of AntiCCP increased the possibility for the diagnosis of RA. However, RF was also moderately related to the diagnosis of RA, and the combination of both tests was more valuable.

## 1. Introduction

The AIHW (2009) defines RA is an autoimmune inflammatory disease that causes joint stiffness, pain—especially in the morning—and reduced bodily function. The illness usually finds symmetrically in both sides of the body, commonly including hand joints. RA affects all the parts of human body, involving many organs, and so is defined as a systemic disease [1]. It also affects the heart, respiratory system, nerves and eyes. The fundamental reason for the disease is not clearly defined. RA attacks individuals in diverse ways and becomes chronic.

There may be stages of reasonable reduction, where symptoms decline noticeably; nonetheless, in the extended term, deprived of helpful treatment, the disease causes disability and impairment [2]. According to Feinstein and Brent (2006), the presence of morning stiffness is a key warning of articular feature. Morning stiffness—such as after getting out of bed, having trouble moving the joints—includes symmetrically both sides of the body part and becomes better with movement [3]. The majority of RA patients suffer with some sort of morning stiffness. Though, using morning stiffness as a tool to differentiate different arthritic conditions is unclear. Additionally, RA is a major reason for early retirement from jobs [4]. Even in the first year after the onset of the disease, economic and social issues for the individual are extreme. Approximately 40 percent of patients are facing heavy difficulty in work within seven years [5]. Almost 60 percent of patients are no longer able to do their professional and personal work after ten years from the onset of the disease per the WHO [6]. Moreover, the sensitivity (54–88%) and specificity (48–92%) of RF assay in its existing manifestation are less than optimal as a diagnostic test [7,8,9,10,11]. Previous studies illustrating the number of antibodies to CCP in mixed groups are moderately sensitive (68%) but highly specific (98%) for RA, comprising patients with infectious diseases, rheumatic diseases and healthy patients [11]. The objective of this study is to assess the sign/symptoms, joints’ involvement, difficulties in daily activities, opportunistic infections and screening accuracy of serology tests of clinically suspected RA patients.

***Novelty of the Paper:***

- Clinically suspect subjects belong to all age groups.
- The present study evaluated sensitivity, specificity, PPV, NPV, LR+ and LR− for individual diagnostic tests along with combinations of different tests.
- Difficulties in daily activities of clinically suspect subjects were shown using a four-point Likert Scale.

## 2. Materials and Methods

Cross-sectional cohort study design was used. Study comprises 290 patients clinically suspected of rheumatoid arthritis. We conducted the study after obtaining ethical clearance from the institutional ethical committee, i.e., IEC/IIMS&R/2019/31. Study subjects were referred from different OPDs of hospitals to UGC Advanced Immunodiagnostic Training and Research Centre for screening. Generally, study subjects came from western Bihar Madhya Pradesh, eastern Uttar Pradesh and Jharkhand, etc. Laboratory persons collected around two milliliters of blood samples from each patient and tested for diagnostic tests RF (Rheumatoid factor), CRP (C-reactive protein) and AntiCCP (Anti-cyclic citrullinated peptide) by using RF-Latex, CRP Latex and ELISA method, respectively. After establishing the rapport with the respondents, the schedule was administered in Hindi and in local dialect. After providing the written consent, the respondents were interviewed personally by the investigator to get firsthand information as well as the real picture of their limbs through direct observation. The study was approved by the appropriate ethics committee. Study subjects were selected in a random manner and were diagnosed according to The 2010 American College of Rheumatology/European League Against Rheumatism’s classification criteria for rheumatoid arthritis’s Classification criteria for RA (score-based algorithm: add scores of categories A–D; a score of 6/10 is needed for classification of a patient as having definite RA) [12].

A. Joint involvement: One *large joint 0 point, 2-10 large joints 1 point, 1-3 small joints (with or without involvement of large joints) 2 points, 4-10 small joints (with or without involvement of large joints) 3 points, >10 joints (at least 1 small joint) 5 points*.

B. Serology (at least 1 test result is needed for classification): *Negative RF and negative ACPA 0 point, Low-positive RF or low-positive ACPA 2 points, High-positive RF or high-positive ACPA 3 points*.

C. Acute-phase reactants (at least 1 test result is needed for classification): *Normal CRP and normal ESR 0 point, Abnormal CRP or abnormal ESR 1 point.*

D. Duration of symptoms: *<6 weeks 0 point, ≥6 weeks 1 point*.

### Statistical Analysis

The responses received through the interview schedule were coded, grouped, processed and tabulated. Data were entered into MS Excel spreadsheet and were analyzed by using SPSS package (16.0 Version). Collected information presented in frequency and percentage. Sensitivity (Sp), specificity (Se), accuracy, positive predictive value (PPV) and negative predictive value (NPV) were calculated for 2010 ACR/EULAR criteria.

## 3. Result

Cross-sectional cohort study design was used. Study comprises 290 patients clinically suspected of RA, aged between 7 to 72 years with mean age 33.54 ± 14.62 (Mean ± SD). Among these, 110 were male and 180 were female clinically suspected patients. Among all study subjects, it was found that 48 study subjects were positive (16.6%). Out of 110 males, 12.7% had RA, and out of 180 females, 18.9% had RA, according to 2010 ACR/EULAR criteria.

Table 1 Illustrates the distribution of study subjects by their health-related issues such as signs and symptoms at the time of data collection. Data clearly depict that most of the study subjects were suffering from tiredness. After that, joint pain (24.8%) shows as the leading problem in RA as compare to other signs and symptoms, i.e., 22.8%. It observed from the findings that those respondents who have ankle swelling (1.7%) were the lowest proportion.

In Table 2, the majority of the study subjects suffered from knee problem, i.e., 61.7%. It is far-flung from other variables in joint involvement. After that, a quantity of leading portion of the study subjects belongs to the criteria of back (28.3%) and finger (25.9%) joints, respectively. Percentage of study subjects suffering with shoulder (18.3%) and hips (17.6%) joints, respectively, followed.

Table 3 depicts the distribution of study subjects by difficulties in daily life activity. Measurement of functional disability was conducted in all subjects at the time of data collection. In the present study, approximately 70% were considered as no difficulty under dressing/grooming such as manipulating laces/buttons of his/her shoes/shirt, and a similar percentage of subjects shows no difficulty in shampoo/oiling hair. Only 0.3% of subjects were unable to do it. Considering those with no difficulties in arising, i.e., standing up from straight chair, and going in/out of bed, both had similar percentages (36%). Whereas concerning difficulties in eating-associated effort, 73.8% of study subjects had no difficulty in cutting vegetables/meat, and 74.8% had no difficulty in lifting a full glass to his/her mouth. The majority of study subjects had no difficulty in walking outdoors on flat ground (54.1%). This was followed by walking on uneven ground (43.4%), climbing five steps up (31%) and going down five step (31%), respectively. In that order, (36.9%) and (31.4%) had some difficulty in walking on flat or uneven ground. Approximately 90% to 96% of the study subjects were not taking help from another person for dressing and grooming, arising, eating and walking.

Sixty-two samples tested positive for AntiCCP, in which 36 had RA as per diagnostic criteria. Whereas 37 clinically suspected patients had RA out of 61 RF positive, and CRP shows 94 samples tested positive which is the highest number of samples among them for CRP. In a combination of serology tests, a majority of study subjects belongs to the group (RF or AntiCCP or CRP) in comparison to only 8.9% belonging to a combination of (RF and AntiCCP and CRP) (Table 4).

In Table 5, the sensitivity, specificity, PPV, NPV, LR+ and LR−, along with a 95% confidence interval of AntiCCP for RA patients, are 75.00%, 88.84%, 57.14%, 94.71%, 86.55%, 6.72, 0.28, respectively. This is compared with RF for RA patients which are 77.08%, 89.67%, 59.68%, 95.18%, 87.59%, 7.46, 0.26, respectively. Sensitivity of RF was higher as compared to AntiCCP.

In Table 6, the distribution on the basis of opportunistic infection shows that age and comorbidities are related to increased risk of opportunistic infection in RA patients.

## 4. Discussion

### 4.1. Presence of Opportunistic Infections (Signs and Symptoms)

According to an arthritis foundation in Atlanta, many people experience fatigue, loss of appetite and a low-grade fever along with pain. The symptoms and effects of RA may come and go. Ongoing high levels of inflammation can cause problems throughout the body [13]. In the present study, study subjects had significant morbidities such as fever, dizziness, tiredness, joint pain, joint swelling, ankle swelling, back pain, muscle pain and neck pain. Synovitis usually develops gradually, often involving the hands, wrists, knees, or feet symmetrically. However, few studies showed even slightly similar results [14,15,16]. Moreover, in the present study, patients suffering with muscle pain was 11.1% and swelling in their ankles was 8.3%.

Increased infection in RA patients can be attributed to a number of factors. First, regardless of treatment, RA is linked to a nearly twofold increased risk of infection when compared to the general population. This is because both the disease and uncontrolled inflammation affect the immune system’s homeostasis. Second, factors such as disease-related problems (immobility, joint surgery) and extra-articular RA symptoms (Felty’s syndrome, rheumatoid lung disease) also have an impact on the likelihood of infections. The possibility of developing bacterial infections in RA patients is independently correlated with older age and comorbidities, particularly cardiovascular disease and diabetes. The comorbidities included a number of chronic conditions, such as dementia, peripheral vascular disease and cerebrovascular disease, that are mostly unrelated to the emergence of opportunistic infections. These are known to be only tangentially associated with opportunistic infections, and their prevalence tends to rise with ageing [17,18].

### 4.2. Joints Involvement of the Study Subjects

RA is a chronic inflammatory disease that causes severe disability [19,20]. RA generally starts with swelling, pain and difficulty in moving finger joints [21]. Another study reported the involvement of small joints of bilateral upper and lower extremities [22]. In the present study, the positivity rate was 41.3% for those who had finger pain.

RA positive rates were much higher in patients with pain in shoulder, neck, back, wrist and toes as compared to patients with no pain.

The present study found that the majority of subjects had knee problem. Some of the patients also had pain in shoulder and hip joints, respectively

The practice of cross-legged sitting in social or religious gatherings was also one of the major causes of knee problem in India. The majority of Indian people usually practice squatting in the toilet. The incapability to do these actions leads to functional disability [20].

### 4.3. Difficulties in Daily Activity of the Study Subjects

In the morning, most of the people have stiff and painful joints, making it tough to begin the day. Everyday tasks including laundry, cleaning, cooking, gardening and entertainment activities can become a challenge as the illness developments [23]. Fries et al. published a Health Assessment Questionnaire–Disability Index (HAQ-DI) in 1980, Pincus et al. published an abridged version (Modified HAQ or MHAQ) after three year and recently Pincus and his team published a multi-dimensional HAQ (MDHAQ) [24,25,26]. The most common feature of functional limitation was walking [43.5% (280/643)], followed by grasping [36.1% (232/643)], fetching [35.5% (228/643)], activity [33.4% (33.4%) 215/643)], personal hygiene [33.0% (212/643)], dressing and grooming [29.7% (191/643)], and getting up [29.1% (187/643)] whereas eating restriction was the least common [27]. A similar finding reported in the present study is that approximately 70% had no difficulty dressing/grooming. No difficulties in arising such as standing up from a straight chair and going in and out of bed had similar percentages (36%). Whereas difficulties in eating-associated effort show 73.8% of study subjects having not any difficulty in cutting vegetables/meat and 23.8% having some difficulty, for lifting a full glass to his/her mouth 66 (22.8%) were showing some difficulty. The majority of study subjects had no difficulty in walking outdoors on flat ground (54.1%). In climbing five steps up or down, (41.7%) in both were showing some difficulties. In addition, a Chinese study reported that a low quality of life and the limitation of joint mobility had great impacts on functional disability in RA patients [28]. Furthermore, another study reported that women reported greater disability than men both in the total HAQ and in the majority of its eight subdimensions [29].

### 4.4. Investigation Profile of the Study Subjects

Out of a total of 290 clinically suspected cases, 61 cases were RF positive, and 62 cases were AntiCCP positive. Among sixty-one RF positive cases, 37 cases were confirmed RA, and among 62 AntiCCP positive cases, 36 cases were confirmed RA as per diagnostic criteria. In addition, several studies reported the diagnostic presentation of AntiCCP on a diversity of investigative stages [30,31]. If the CRP level is high such as the ESR, it shows signs of pain, swelling and redness [32]. In the present study of 290 clinically suspected cases, 94 cases were acute phase reactant CRP positive. Among 94 CRP-positive cases, 45 cases were confirmed RA as per diagnostic criteria. If considering combinations of tests (RF, AntiCCP and CRP), it was detected that 100 percent of cases were confirmed RA as per diagnostic criteria. Identification of RF as well as AntiCCP is valuable for evaluation of RA, and, if measured simultaneously, combinations of tests (RF and AntiCCP) showed higher a prediction of RA. Regardless of the higher diagnostic assessment of AntiCCP and RF, there is a need for new serological biomarkers to advance the early diagnosis of RA. When CRP was used unaided or in combination with other well-known indicators, this can help in the early diagnosis, estimation of disease and assessment of response to treatment in RA patients [33]. Previous studies reported that RF showed less specificity for RA and AntiCCP tests showed higher sensitivity and specificity as compared to RF tests [34]. The present study showed specificities of RF and AntiCCP were 89.67% and 88.84 for RA, respectively. This present an outcome in contrast with other studies. In addition, RF had a slightly higher sensitivity than the AntiCCP test. Specificity showed decline trends, if considering a combination of both RF and AntiCCP auto-antibodies for the diagnosis of RA.

## 5. Conclusions

The present study found substantiation of a majority of RA patients had knee problems in an Indian context. The identification of AntiCCP and RF is valuable for the diagnosis of RA, and, if measured simultaneously, combinations of tests (RF and AntiCCP) showed a higher prediction of RA. CRP may also be valuable as an indicator of early inflammation in clinically suspected RA patients. Identification of the disease at an early phase will benefit the associated daily activity of the study subjects.

## Figures and Tables

**Table 1:** Distribution of signs and symptoms profile of the study subjects.

Sign/Symptom	Study Subjects with Signs and Symptoms n (%) (n = 290)	Study Subjects RA Positive according to 2010 ACR/EULAR Criteria (n = 48)
Fever	57 (19.7)	17
Dizziness	26 (8.9)	13
Tiredness	72 (24.8)	33
Joint Pain	66 (22.8)	21
Joint Swelling	57 (19.7)	17
Ankle Swelling	5 (1.7)	4
Back Pain	46 (15.9)	15
Muscle Pain	18 (6.2)	2
Neck Pain	18 (6.2)	6

**Table 2:** Distribution of joints’ involvement for the study subjects.

Involved Joints	Study Subjects with Discomfort n (%) (n = 290)	Study Subjects RA Positive according to 2010 ACR/EULAR Criteria (n = 48)
Finger	75 (25.9)	31
Wrist	60 (20.7)	33
Toes	18 (6.2)	10
Shoulder	53 (18.3)	16
Neck	35 (12.1)	11
Back	82 (28.3)	21
Elbow	18 (6.2)	8
Ankle	43 (14.8)	17
Knee	179 (61.7)	40
Hips	51 (17.6)	22

**Table 3:** Distribution of study subjects by difficulties in daily activities.

Daily Activities	No Difficulty	Some Difficulty	Much Difficulty	Unable to Do
***Difficulties in Dressing and Grooming:***				
Shoelaces and Buttons	204 (70.3)	79 (27.2)	6 (2.2)	1 (0.3)
Shampoo/Oiling hair	205 (70.7)	78 (26.9)	6 (2.1)	1 (0.3)
***Difficulties* *in Arising:***				
Stand up from a Straight Chair	106 (36.6)	102 (35.2)	77 (26.6)	5 (1.6)
Get in and out of Bed	105 (36.3)	103 (35.5)	77 (26.6)	5 (1.6)
***Difficulties in Eating:***				
Cut Vegetables/Meat	214 (73.8)	69 (23.8)	5 (1.7)	2 (0.7)
Lift a Full Glass to Mouth	217 (74.8)	66 (22.8)	5 (1.7)	2 (0.7)
***Difficulties* *in Walking:***				
Walk Outdoors on Flat Ground	157 (54.1)	91 (31.4)	38 (13.1)	4 (1.4)
Climb Five Steps up	90 (31.0)	121 (41.7)	73 (25.2)	6 (2.1)
Go down Five Steps	90 (31.0)	121 (41.7)	73 (25.2)	6 (2.1)
Walk on Uneven Ground	126 (43.4)	107 (36.9)	53 (18.3)	4 (1.4)
***Need Help from Another Person for:***				
Dressing and Grooming	272 (93.8)	15 (5.2)	3 (1.0)	0 (0.0)
Arising	273 (94.1)	15 (5.2)	2 (0.7)	0 (0.0)
Eating	280 (96.6)	8 (2.7)	2 (0.7)	0 (0.0)
Walking	259 (89.3)	26 (9.0)	5 (1.7)	0 (0.0)

**Table 4:** Distribution of study subjects by serology test results.

Serology Test	Study Subjects with Test Positive n (%) (n = 290)	Study Subjects with RA Positive according to 2010 ACR/EULAR Criteria (n = 48)
RF	61 (21.0)	37
AntiCCP	62 (21.4)	36
CRP	94 (32.4)	45
CRP or AntiCCP	112 (38.6)	47
CRP and AntiCCP	39 (13.4)	32
RF or AntiCCP	95 (32.8)	48
RF and AntiCCP	27 (9.3)	25
RF or CRP	106 (36.5)	46
RF and CRP	46 (15.9)	34
RF or AntiCCP or CRP	128 (44.1)	48
RF and AntiCCP and CRP	26 (8.9)	23

**Table 5:** Sensitivity, specificity, PPV, NPV, LR+ and LR−.

Serology Test	Sensitivity (%) 95% CI	Specificity (%) 95% CI	Positive Predictive Value (%) 95% CI	Negative Predictive Value (%) 95% CI	Accuracy (%)	Positive Likelihood Ratio 95% CI	Negative Likelihood Ratio 95% CI
RF	77.08 (62.68–87.95)	89.67 (85.13–93.20)	59.68 (46.45–71.95)	95.18 (91.53–97.56)	87.59	7.46 (4.99–1.15)	0.26 (0.15–0.43)
AntiCCP	75.00 (60.40–86.35)	88.84 (84.18–92.52)	57.14 (44.05– 69.54)	94.71 (90.95–97.24)	86.55	6.72 (4.55–9.94)	0.28 (0.17–0.46)
CRP	93.75 (82.78–98.62)	78.51 (72.80–83.52)	46.39 (36.20–56.81)	98.45 (95.52–99.66)	81.03	4.36 (3.39–5.61)	0.08 (0.03–0.24)
CRP+ AntiCCP	97.92 (88.88–99.65)	72.31 (66.22–77.85)	41.23 (32.09–50.83)	99.43 (96.86–99.90)	76.55	3.54 (2.87–4.35)	0.03 (0.00–0.20)
RF+ AntiCCP	100 (92.53–100)	80.99 (75.47–85.73)	51.06 (40.54 –61.52)	100 (98.12–100)	84.14	5.26 (4.06–6.82)	0.00 -
RF + CRP	93.75 (82.78–98.62)	72.73 (66.65–78.24)	40.54 (31.32–50.27)	98.32 (95.17–99.63)	76.21	3.44 (2.76–4.28)	0.09 (0.03–0.26)
RF+ AntiCCP + CRP	100 (92.53–100)	66.12 (59.78–72.05)	36.92 (28.63–45.83)	100 (97.70–100)	71.72	2.95 (2.47–3.52)	0.00 -

**Table 6:** Distribution of study subjects on the basis of opportunistic infection.

Age Group (Years)	Co-Morbid n (%)	Bacterial n (%)	Viral n (%)	Fungal n (%)
≤20 (n = 66)	-	-	10 (15.15%)	3 (4.54%)
21–40 (n = 138)	10 (7.24%)	-	-	8 (5.79%)
41–60 (n = 77)	50 (64.93%)	11 (14.28%)	-	-
>60 (n = 9)	9 (100.0%)	4 (44.44%)	2 (22.22%)	1 (11.11%)

## Data Availability

Not applicable.
